# Point-of-care assay for drunken driving with Pd@Pt core-shell nanoparticles-decorated ploy(vinyl alcohol) aerogel assisted by portable pressure meter

**DOI:** 10.7150/thno.42601

**Published:** 2020-04-06

**Authors:** Yu Zhang, Quanyi Liu, Chong-Bo Ma, Qingqing Wang, Meiting Yang, Yan Du

**Affiliations:** 1 State Key Laboratory of Electroanalytical Chemistry, Changchun Institute of Applied Chemistry, Chinese Academy of Sciences, Changchun, Jilin 130022, China.; 2 Department of Chemistry, University of Science & Technology of China, Hefei, Anhui 230026, China; 3 Key Laboratory of Nanobiosensing and Nanobioanalysis at Universities of Jilin Province, Key Laboratory of Polyoxometalate Science of Ministry of Education, National & Local United Engineering Laboratory for Power Batteries, Department of Chemistry, Northeast Normal University, Changchun, Jilin Province 130024, China

**Keywords:** point-of-care, Pd@Pt core-shell nanoparticles, poly(vinyl alcohol) aerogel, alcohol sensing, portable pressure meter

## Abstract

Alcohol abuse causes health problems and security accidents. A reliable and sensitive detection system for alcohol has been an instinctive demand in law enforcement and forensic. More efforts are demanded in developing new sensing strategy preferably with portable and non-invasive traits for the pushforward of point-of-care (POC) device popularization.

**Methods**: We developed a POC diagnosis system for alcohol assay with the aid of alcohol oxidase (AOX) pre-joining in the system as well as Pd@Pt core-shell nanoparticles (abbreviated to Pd@Pt) that were decorated on ploy(vinyl alcohol) aerogel with amphiphilicity. Biological samples like saliva and whole blood can be absorbed by the aerogel in a quick process, in which the analyte would go through a transformation from alcohol, H_2_O_2_, to a final production of O_2_, causing an analyte dose-dependent signal change in the commercial portable pressure meter. The cascade reactions are readily catalyzed by AOX and Pd@Pt, of which the latter one possesses excellent peroxidase-like activity.

**Results**: Our design has smartness embodied in the aerogel circumvents the interference from methanol which is more ready to be catalyzed by AOX. Under the optimal conditions, the limit of detection for alcohol was 0.50 mM in saliva, and is able to distinguish the driving under the influence (DUI) (1.74 mM in saliva) and driving while impaired (DWI) (6.95 mM in saliva) in the national standard of China.

**Conclusion**: Our proof-of-concept study provides the possibility for the establishment of POC device for alcohol and other target detection, not only owing to the sensing qualification but also thanks to the architecture of such sensor that has great flexibility by replacing the AOX with glucose oxidase (GOX), thenceforth realizing the accurate detection of glucose in 0.5% whole blood sample. With the advantages of easy accessibility and anti-interference ability, our sensor exhibits great potential for quantitative diagnostics in biological system.

## Introduction

Unsafe alcohol consumption is one of the major causes of traffic accidents, violence and alcoholic liver disease 1-3. Such accidents and health problems caused by drinking have received more and more attention. Traditional in-lab detection of alcohol methods include gas chromatography 4, flow injection analysis 5, and infrared detection 6, almost non-exceptionally all of which use cumbersome and costly equipment in spite of their high accuracy. There is an imminent and unmet need for the development of effective diagnostic tests to provide reliable and convenient assessment for alcohol abuse 7, 8. In the case of driving under influence (DUI) testing, the utilization of disposable sample is always preferred, like the exhalation detected by breathanalyzers. However, the accuracy is affected by local temperature, humidity, environmental fumes and other factors of human subjects 9. Sometimes repeated measurements are needed to raise credibility. The alcohol level in blood is a golden standard for determination of drunken driving, nevertheless, the sample taking process is usually accompanied with pain and inconvenience 10. The alternative choice for identification of alcohol intoxication is to test the alcohol concentration in saliva, which is much easier to collect. Thus, it is crucial to develop a rapid, accurate and convenient method to detect saliva alcohol concentration.

The materials of aerogel, with super-absorbing capacity, also has many other unique characteristics like lightweight, high porosity and large surface area 11, enabling broad applications like sensors, drug delivery, thermal and acoustic insulations, energy storage as well as super-absorbents 12-16. Among the aerogel family, the one formed with a straightforward route, preferably originated from a low-cost raw material is strongly desired for saliva sample collection. Our recent study has demonstrated a simple synthesis method for ploy(vinyl alcohol) (PVA) based aerogels, which had hydrophobic surface though, could be easily transformed into amphiphilic monoliths by a modified synthesis method where maleic acid is introduced. As a result, the aerogel can work as saliva sample collector for alcohol detection. In order to realize the portable detection of alcohol, the next affair that needs to be considered is to transduce the target into readable signal. As known to all, signal transduction, is always realized by formation of certain immune complex or a distinct chemical reaction to arouse changes in electrochemical signal, fluorescence emission, or even color appearing 17-29. Therefore, the rate of the reaction that stimulates the signal change is the key for swift detection. In biological system, the metabolism process of alcohol relies on the enzymatic reaction 30. Inspired by the this, people have already been familiarized with a sequence of transformation processes of alcohol, one of which is the conversion process of alcohol→hydrogen peroxide (H_2_O_2_)→oxygen (O_2_) with the first procedure catalyzed by alcohol oxidase enzyme (AOX) and the second by catalase (CAT) 31.

**Figure 1:**
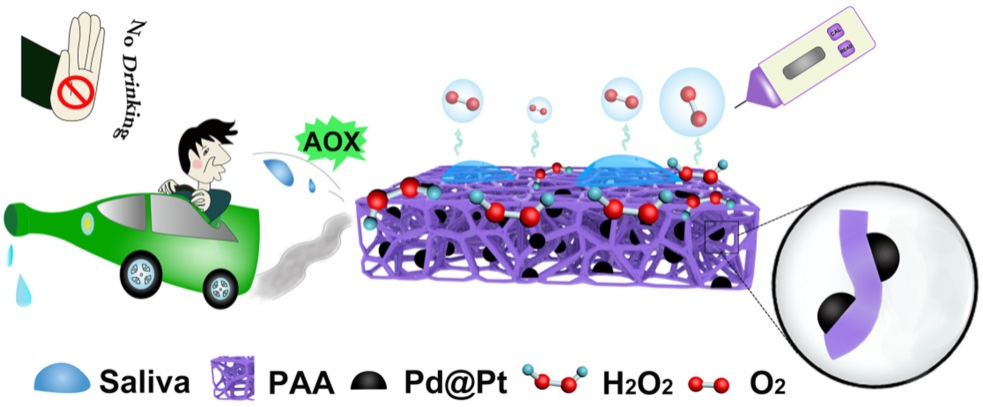
Schematic illustration of PAAC with CAT-like activitiy and alcohol detection with portable pressure meter in POC assay.

Hence, in this work, we functionalized the PVA-based amphiphilic aerogel (PAA) by anchoring the Pd@Pt core-shell nanoparticles (denoted as Pd@Pt) with high CAT-mimicking property 32-36, which converted alcohol detection to gas pressure signals in a sealed device and could be quantitatively detected with portable pressure meter, by virtue of the cascade reactions catalyzed by AOX that pre-existed in the solution (Figure 1). Here, the aerogel functions as a highly biocompatible sponge-like absorbent that can be used as biological sample collector. The benefits brought from the aerogel is not only confined to the richness in oxygenated groups that facilitates the uniform nucleation of the modifiers on aerogel surface, but also the amphiphilic surface encourages transportation of the substrate and product through the aerogel matrices 37. Additionally, the intrinsic oxygenated groups like -COOH and -OH groups on the aerogel surface pose high affinity with highly polar methanol molecule by virtue of hydrogen bonding, thus greatly slower the decomposition of methanol catalyzed by AOX. The anchored Pd@Pt as an efficient nanozyme contributed its excellent CAT-like activity and POD-like activity 38-48. More importantly, the decomposition of H_2_O_2_ into H_2_O and O_2_ are fast, robust and environmentally friendly, which is suitable for POC testing 49-54. As a proof of concept, the proposed PAA/Pd@Pt composite (denoted as PAAC) can also work as glucose sensor by replacing the AOX to glucose oxidase (GOX). The incorporation of modified aerogel and the pressure meter may be considered as a promising tool for rapid detection of alcohol and glucose with the duration of sample preparation about 30 min and signal producing step about 15 min, and it provides a new way of signal transduction for alcohol and glucose detection by establishing a model as portable universal sensor.

## Materials and Methods

### Chemicals and materials

Poly(vinyl alcohol) (PVA, M_w_ ~195000), maleic acid (MA) and ascorbic acid were purchased from Aladdin Reagent Company (Shanghai, China). Chloroplatinic acid hexahydrate (H_2_PtCl_6_∙6H_2_O) and palladium chloride (PdCl_2_) were bought from Shanghai Chemical Reagent Co., Ltd. (Shanghai, China). Alcohol oxidase enzyme (AOX) extracted from *Pichia pastoris* (10-40 units/mg protein), glucose oxidase (GOX), 2-(N-morpholino)-ethanesulphonic acid (MES, ≥99%), potassium acetate (KAc) and Pluronic F-127 were supplied by Sigma Chemical Co. (St. Louis, MO, USA). Hydrogen peroxide (H_2_O_2_, 30%), hydrochloric acid and sulfuric acid were obtained from Beijing Chemical Works (Beijing, China). 3,3,5,5-tetramethylbenzidine (TMB, >99.0%) was purchased from Shanghai Macklin Biochemical Co., Ltd. (Shanghai, China). The double deionized water (hereinafter denoted as H_2_O for short) was used in all experiments (Millipore water purification system, Merck, Darmstadt, Germany). All the chemicals and reagents were used without further purification.

### Apparatus and characterization

Scanning Electron Microscopy (SEM) images and Energy-dispersive X-ray spectroscopy (EDX) elemental mapping were performed on an XL-30 ESEM FEG instrument (FEI, Hillsboro, Oregon, USA). Transmission electron microscaopy (TEM) images were performed on a Talos F200S instrument (FEI, Hillsboro, Oregon, USA) with an acceleration voltage of 200 kV. Frozen-drying process was completed in Alpha 1-2LD plus instrument (Marin Christ, Osterode, Germany). Fourier-transform infrared spectroscopy (FT-IR) spectra was obtained on VERTEX 70 (Bruker, Karlsruhe, Germany). Thermogravimetric analysis (TGA) was conducted in air atmosphere from 25 to 800 ^o^C on SDT Q600 (TA, Wilmington, Delaware, USA). X-ray photoelectron spectroscopy (XPS) was investigated using an ESCALAB-MKⅡ spectrometer (VG Co., United Kingdom) with Al Kα X-ray radiation as the X-ray source for excitation. Ultraviolet-visible (UV-Vis) absorption measurements were collected on a Spark^TM^ Multimode Microplate reader (Tecan, Männedorf, Switzerland). The steady-state kinetics were performed on NanoDrop OneC (Thermo Fisher Scientific, Wilmington, DE, USA). Gas pressure values were measured by a portable pressure meter (PASSTECH, Xiamen, China).

### Synthesis of PAA

PAA was synthesized through a simple hydrothermal method. In brief, in PVA solutions with concentration of 5%, 6%, 7% and 8% (weight percent (wt%)), certain amount of MA was added, with molar ratio of carboxyl groups in MA to hydroxyl groups in PVA of 0.3, 0.4 and 0.5 (C/H=30%, 40% and 50%). After complete dissolution, 1 mL concentrated sulfuric acid was dropped into the solution slowly and the mixture was stirred for 30 min. Then the solutions were transferred into Teflon-lined stainless-steel autoclaves and maintained at 200^ o^C for 24 h. The obtained products were immersed in H_2_O to remove the impurities. The final PAA products were obtained after lyophilization. The product synthesized with 5 wt% PVA with C/H of 40% was named as 5-PAA-40. In a similar fashion, other samples were named as M-PAA-N (M and N represent the concentration of PVA and C/H value, respectively).

### The *in-situ* modification of Pd@Pt on aerogel surface

Thirty milligrams of M-PAA-N was incubated with the mixture containing 1.8 mL of H_2_PtCl_6_ (80 mM), 0.4 mL of H_2_PdCl_4_ (40 mM, prepared by HCl and PdCl_2_), 0.8 mL of Pluronic F-127 (25 mg/mL) and 2 mL of ascorbic acid (200 mM) and shaken for 4 h. Then the mixture was transferred into a Teflon-lined stainless-steel autoclaves and kept at 90^ o^C for 2 h. A series of PAA/Pd@Pt composite (PAAC) were synthesized under M and N values and the corresponding samples are denoted as M-PAAC-N. The final products were prepared by lyophilization after being immersed in H_2_O to remove the ions and other impurities. For comparison, aerogels modified by monoplasmatic Pt and Pd (denoted as Pt-PAA and Pd-PAA) were also synthesized under the same conditions except for the absence of H_2_PdCl_4_ or H_2_PtCl_6_, respectively.

### The CAT- and POD-like activity of PAAC

The CAT-like activity was assessed by measuring gas pressure values of H_2_O_2_ decomposition using a portable pressure meter. 0.2 mL of various concentrations of H_2_O_2_ and 1.0 mg of M-PAAC-N were incubated in phosphate buffer solution (pH 7.4) at 37^ o^C for 15 min. H_2_O_2_ was decomposed into oxygen with pressure increased in a confined space. The concentration of H_2_O_2_ was evaluated by directly measuring the gas pressure values.

The POD-like activity assays were carried out in a reaction volume of 1.0 mL MES-AC buffer solution (10 mM, pH 4.0) containing 1.0 mg of M-PAAC-N, 1.0 mM H_2_O_2_ and 0.8 mM TMB as substrate, and monitoring the absorbance at 652 nm within a certain time by NanoDrop OneC.

### Detection of alcohol by pressure meter

Alcohol detection was performed as follows: A 8 µL aliquot of AOX (12 U) and 292 µL of different concentrations of alcohol were incubated in phosphate buffer solution (pH 7.4) at 37^ o^C for 30 min. 1.5 mg of 6-PAAC-30 were added into the above solutions. The mixtures were incubated at 37^ o^C for 15 min in a confined space. The needle of pressure meter was inserted into the rubber-sealed wells of a 96-well plate. The gas pressure values were read within 3 s.

The saliva samples were collected early morning after the oral cavity of the volunteer was thoroughly washed with H_2_O. The saliva samples were diluted 2.5-fold for further use. Six drinking volunteers drank different amounts of liquor and beer. First, the alcoholism of volunteers was detected on breathanalyzer. If the result was positive, saliva sample was collected for pressure meter testing.

### Generalization test

Glucose detection was performed as follows: A 50 µL aliquot of GOX (20 mg /mL) and 150 µL of different concentrations of glucose were incubated in phosphate buffer solution (pH 7.0) at 37^ o^C for 30 min. Then, 1.0 mg of 6-PAAC-30 were added into 200 µL of the above glucose solution, then incubated at 37^ o^C for 15 min in a confined space. Gas pressure values were recorded. The whole blood of a healthy mouse was diluted 200-fold with phosphate buffer solution (pH 7.0) to decrease the matrix effect. The spiked samples were detected by both glucose meter and pressure meter.

### Cytotoxicity tests

The relative viability of cells was determined by MTT (3-(4,5-dimethylthiazolyl)-2,5-diphenyltetrazolium bromide) assay. The HaCat cells were seeded in a 96-well plate at a density of 10000 cells per well for 24 h. Then 1.0 mg of 6-PAAC-30 was added to the culture medium and incubated for 24 h. To determine the toxicity, 20 µL of MTT (5 µg/mL) solution was added to each well and cultured at 37 ^o^C for 4 h. The media was removed and 150 µL of DMSO was added to each well. After shaking for 10 min, the absorbance at 492 nm were determined by Spark^TM^ Multimode Microplate reader (Tecan, Switzerland). Three parallel measurements were carried out for the cell viability of 6-PAAC-30.

## Results and Discussion

### Synthesis and Characterization of PAAC

**Figure 2:**
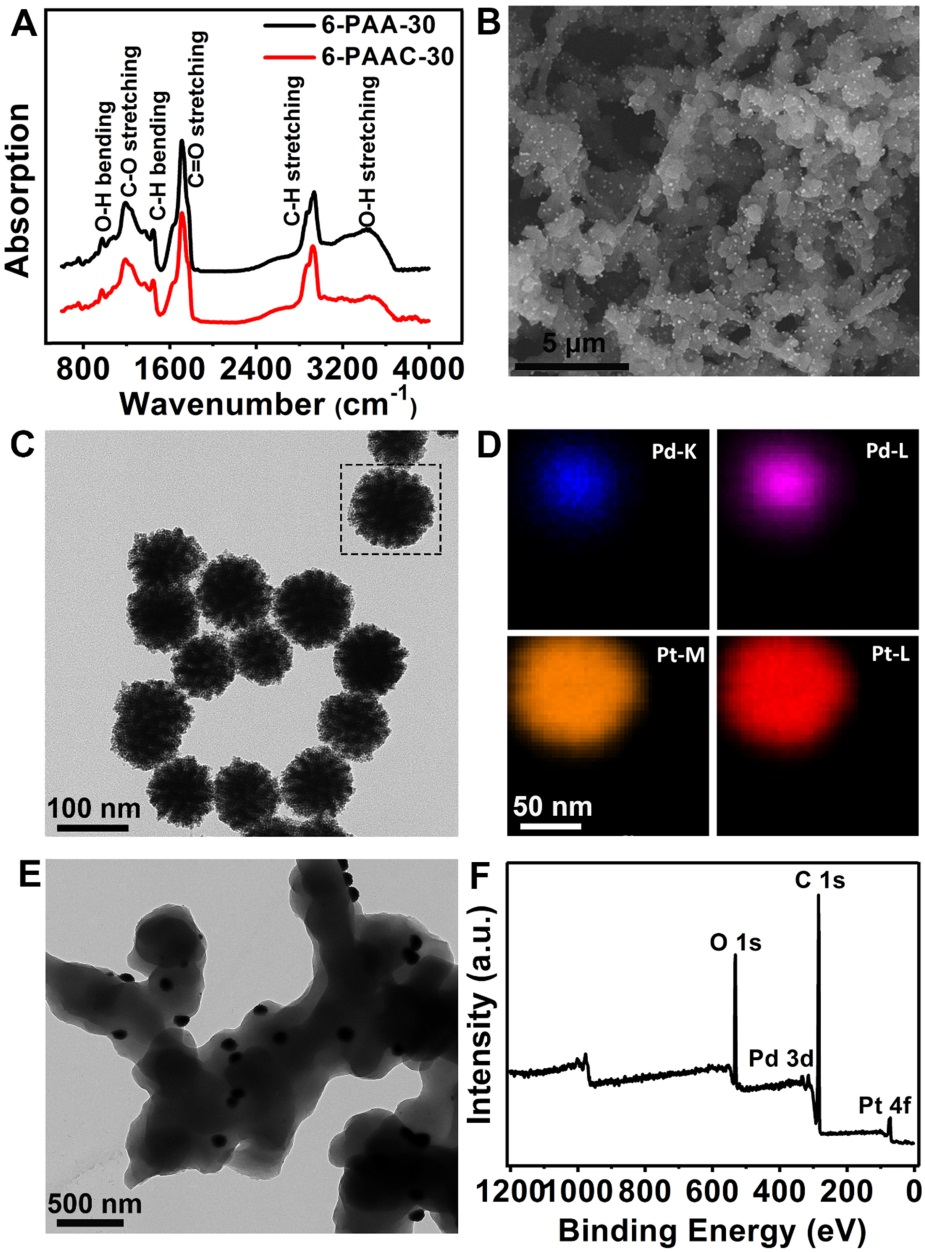
** (A)** FT-IR spectra of 6-PAA-30 and 6-PAAC-30. **(B)** SEM image of 6-PAAC-30. **(C)** TEM image and **(D)** corresponding elemental mappings of Pd@Pt. **(E)** TEM image of 6-PAAC-30. **(F)** XPS spectrum of 6-PAAC-30.

The PAA works as an eligible substrate for Pd@Pt growth. As a typical example, 6-PAA-30 was first synthesized and characterized by FT-IR (Figure 2A). The peaks at 1200 cm^-1^ and 1715 cm^-1^ correspond to C-O stretching and C=O stretching, respectively, indicating the existence of ester bonds formed by the esterification reaction between -COOH and -OH. The peaks at 1450 cm^-1^ and 2920 cm^-1^ are attributed to C-H bending and -CH_2_ stretching. It has been proved by a previous report that the oxygen-containing groups on the substrate, especially -OH, dominant the nanoparticles nucleation and growth process on its surface 55. This conclusion also supports the eligibility of PAA as a substrate for Pd@Pt nucleation and growth. A broad absorption band between 3300 cm^-1^ and 3700 cm^-1^ resulted from O-H stretching is also visible, laying the groundwork for *in-situ* modification of Pd@Pt on the surface. It was realized by a simple percolation method in a hydrothermal process in which the pre-formed 6-PAA-30 were immersed in the solution containing Pt^ 4+^ and Pd^2+^ ions. The band between 3300 cm^-1^ and 3700 cm^-1^ decreased its intensity after modification of Pd@Pt, maybe owing to the reduction effect brought by ascorbic acid as well as the occupation of oxygenated groups for nucleation of Pd@Pt 55-57. Highly dense of Pd@Pt were uniformly distributed on the surface of 6-PAA-30 which has dendritic structure (Figure 2B). The free-standing Pd@Pt have average diameter of ~100 nm (Figure 2C). Element mappings (Figure 2D) insure their core-shell structure, where Pd forms the inner core and Pt exhibits uniform dendritic growth on Pd surface, which is further confirmed by the EDS line scans (Figure S1, in Supporting Information (SI)) for a single particle. The two regions highlighted in gray indicate the ~20 nm thickness of the Pt shell, almost consistent with Figure 2D. In the high-resolution TEM image, the evident contrast between the Pd@Pt and the 6-PAA-30 further insures the surface modification (Figure 2E). The XPS (Figure 2F) and EDX (Figure S2, in SI) characterizations further confirm the chemical composition of the product, of which the former one suggests co-existence of C, O, Pt and Pd elements. Four predominant peaks at 532.0, 337.5, 285.0 and 72.6 eV can be found in XPS spectra, corresponding to O 1s, Pd 3d, C 1s and Pt 4f, respectively. All these results confirmed the successful synthesis of 6-PAAC-30.

### The CAT-like activity of PAAC

**Figure 3:**
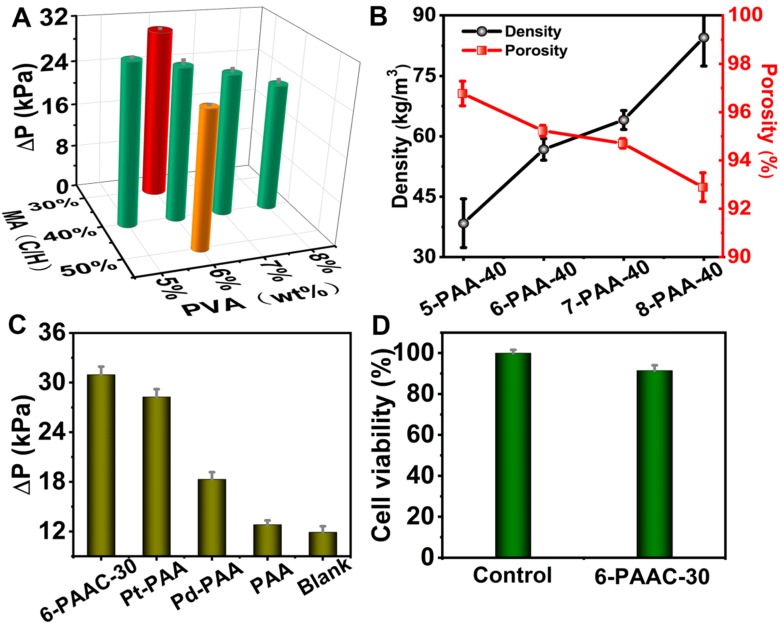
** (A)** Comparison of the CAT-like activities of M-PAAC-40 (M was 5%, 6%, 7% and 8%) and 6-PAAC-N (N was 30%, 40% and 50%). **(B)** Density and porosity of 5-PAA-40, 6-PAA-40, 7-PAA-40 and 8-PAA-40. **(C)** Comparison of the CAT-like activities of different catalysts by measuring ΔP with 20 mM H_2_O_2_ in phosphate buffer (pH 7.4) at 37 ^o^C after reaction for 15 min. **(D)** The cell viability of the 6-PAAC-30 incubated with HaCat cells. Note: Three parallel measurements were carried out in this and all the following figures to obtain the mean values and error bars.

The CAT-like activity was expressed in catalysis for transformation process of H_2_O_2_ into O_2_. Theoretically, under standard conditions, 1 mmol H_2_O_2_ decomposition is responsible for 11.2 mL O_2_ generation 58. The gas generation leads to obvious pressure increases in a sealed device and detected by a portable pressure meter. The pressure change (ΔP), defined as the pressure difference before and after reaction under a specified reaction time, was positively correlated to the production of O_2_. In order to optimize the PVA concentration, we fixed the C/H value at 40% and altered PVA concentration. Four samples of 5-PAAC-40, 6-PAAC-40, 7-PAAC-40 and 8-PAAC-40 were synthesized with the same amount of precursors addition for Pd@Pt, whose SEM images are shown in Figure S3 (in SI). All of the samples have well-dispersed Pd@Pt on surface. The loading amount (LA) was calculated by TGA tests to be 10.5%, 8.28%, 6.12% and 4.53%, respectively, with ΔP values decreased in turn, indicating the gradually attenuated CAT-like activity (Figure 3A). Similar phenomenon was found for POD-like activity (Figure S4A, in SI). There was an obvious trend that the crosslinking density is increased with the increasing PVA concentration, meanwhile the porosity is decreased (Figure 3B). The LA is inversely proportional to the crosslinking density. Although 5-PAAC-40 exhibited the best catalytic performance, the over-loose structure was unfavorable for biological sample collection. Hence, the aerogel made from 6% PVA solution was considered as the most advantageous substrate and 6-PAA-40 was considered as the optimal choice as a trade-off between the mechanical strength and the performance. In fact, the mass of MA also affects the density of the aerogel, therefore, samples varying the mass of MA were prepared (6-PAAC-30, 6-PAAC-40 and 6-PAAC-50), whose LA of the Pd@Pt were 13.1%, 8.28% and 4.85%. The CAT-like activity (Figure 3A) and POD-like activity (Figure S4B, in SI) both decreased from 6-PAAC-30 to 6-PAAC-50. Therefore, 6% PVA solution with 30% -OH groups theoretically occupied by MA was employed as the optimal condition. The synergistic effect of Pt and Pd was reflected in the best performance of 6-PAAC-30 in comparison with single metal modified counterparts (Figure 3C and Figure S4C, in SI). Furthermore, the reusability of our aerogel catalyst was investigated, the sample of 6-PAAC-30 maintained 84.9% of the maximized activity even under ten cycles of reuse (Figure S5, in SI). In addition, this POC diagnosis touches on the collections of saliva to realize non-invasive measurement of drunken driving. Thus, it necessary to investigate the cytotoxicity of PAAC. HaCat cells were used to testify the biocompatibility, which was witnessed by a high survival rate of 91.5% even though 1.0 mg of 6-PAAC-30 was exposed to the cells for 24 h (Figure 3D). The excellent biocompatibility benefits from the low-toxicity precursors and the effective fixation for the heavy metal Pt and Pd avoiding the toxicity under free state.

### Optimization of assay conditions

**Figure 4:**
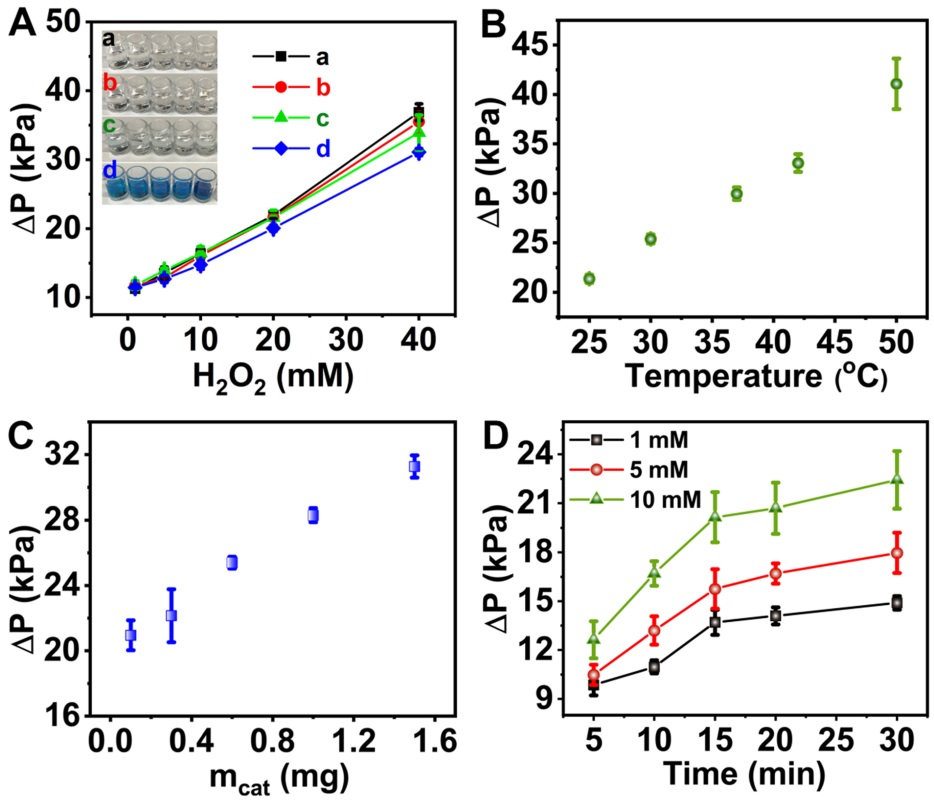
** Pressure change profiles for the H_2_O_2_ decomposition reaction. (A)** effect of the pH with H_2_O_2_ ranging from 1 mM to 40 mM at pH 7.4 in the absence of TMB (a, black dots) and in the presence of TMB (c, green dots) or pH 4.0 in the absence of TMB (b, red dots) and in the presence of TMB (d, blue dots). Inset was the images of reaction solutions at pH 7.4 or pH 4.0 in the presence and absence of TMB. **(B)** effect of the temperature. **(C)** effect of the catalyst dosage. **(D)** effect of the reaction time with varied concentration of H_2_O_2_.

To achieve optimal outcome, the influence of pH, temperature, catalyst dosage, reaction time and H_2_O_2_ concentration were systematically investigated. The change in pH posed no significant effect on the CAT-like activity of 6-PAAC-30 since no evident difference in ΔP values was found in pH 7.4 and pH 4.0 buffer solutions when H_2_O_2_ ranged from 1 to 40 mM (Figure 4A, black and red dots). After the addition of 1.2 mM TMB, ΔP values were decreased by 12.4% in pH 4.0 buffer solution (Figure 4A, green dots) and 8.13% in pH 7.4 buffer solution (Figure 4A, blue dots), respectively. It was because the transformation from TMB to its oxidized state (oxTMB) consumed some H_2_O_2_ that would have produced O_2_. Another noticeable phenomenon is the color change from pale to blue in pH 4.0, which did not happen in pH 7.4 (Figure 4A, inset). A conclusion was drawn that little oxTMB was formed under pH 7.4, suggesting an inhibited POD-like activity under such condition 59-61. An inference from such phenomena may be formulated that the CAT-like activity dominates the H_2_O_2_ decomposition mechanism (from H_2_O_2_ to O_2_) under pH of 7.4 hence such a pH value was adopted in further experiments, serving for the pressure meter readout. Moreover, such a pH is close to the one in human saliva, and is favorable to the detection in biological samples 62, 63. The catalytic performance was enhanced at elevated temperature and higher catalyst dosage (Figure 4B and C). We varied the concentration of H_2_O_2_ from 1 mM to 10 mM and found that a reaction time of 15 min allows the reaction to an almost completion (Figure 4D). Thus, the reaction time was determined to be 15 min based on the concentration of H_2_O_2_ according to the time needed for a specific reaction to be complete.

### Detection alcohol using 6-PAAC-30

**Figure 5:**
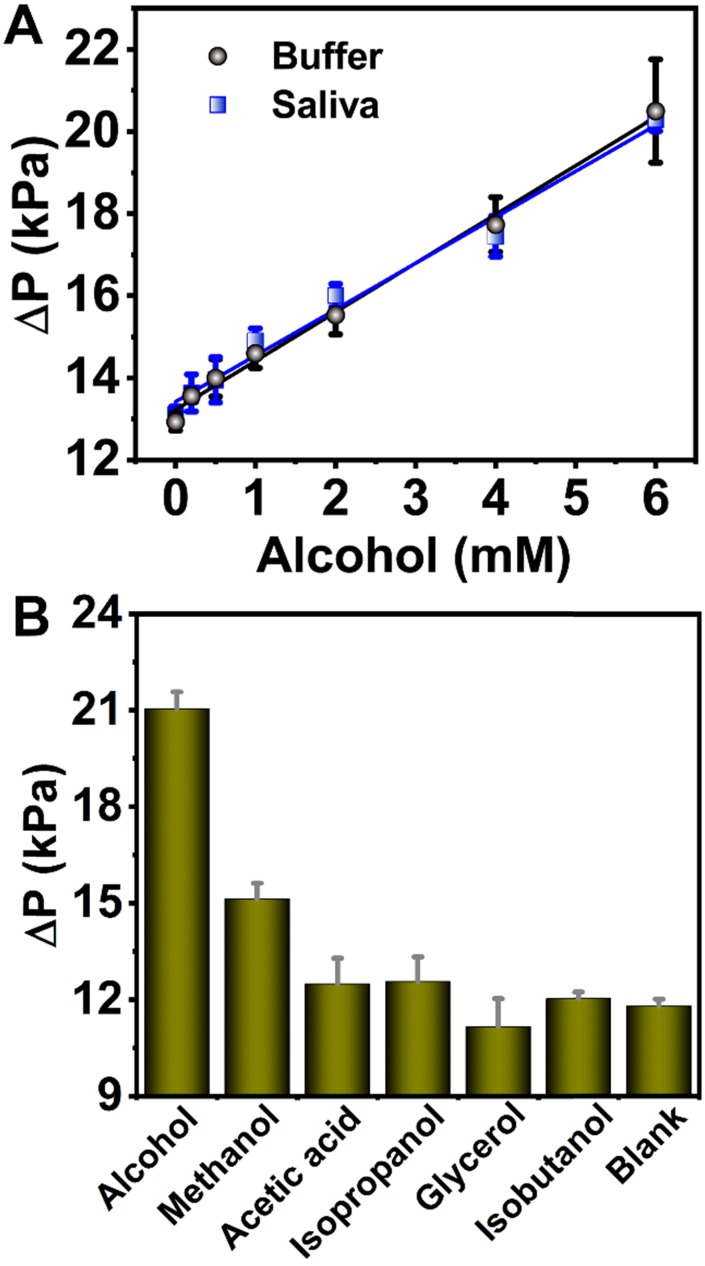
**(A)** The linear correlation between ΔP and alcohol concentration in the 0-6 mM range in buffer (black dots) and in 40% saliva (blue dots). Quantity of enzymes: AOX, 12 U; 6-PAAC-30, 1.5 mg. **(B)** Selectivity analysis for alcohol detection. The analyte concentrations were all 20 mM. Quantity of enzymes: AOX, 7.5 U; 6-PAAC-30, 1.0 mg.

The alcohol concentration is embodied in the ΔP values that are H_2_O_2_ concentration-dependent. As we know, 1 mmol of ethanol decomposes, producing 1 mmol of H_2_O_2_ under the catalysis of AOX. In consideration of the detection range, 15 min reaction time was chosen in biological sample tests. The ΔP values under different alcohol concentration (0-6 mM) were investigated for acquisition of the detection limit (Figure 5A). The linear response for alcohol was fitted with the equation of ΔP = 1.19 C_Alcohol_ + 13.2 (R^2^ = 0.994). The limit of detection (LOD) was determined to be 0.50 mM via the 3σ rule. 40% saliva sample obtained a standard curve ΔP = 1.12 C_Alcohol_ + 13.4 (R^2^ = 0.985) with the LOD of 0.54 mM (Figure 5A). A recovery rate of 94.1% was obtained for 40% saliva from the slope of the two curves thus a detection limit of 1.35 mM in 100% saliva was calculated (Figure S6, in SI), which is lower than the national standard ((People's Republic of China Public Safety Industry Standard, GA/T 843-2009) for “driving under the influence (DUI)” (1.74 mM in saliva) and “driving while impaired (DWI)” (6.95 mM in saliva).

The possible interference alcohol analogues like acetic acid, isopropanol, glycerol and isobutanol were employed in the specificity tests (Figure 5B). The alcohol oxidase extracted from *Pichia pastori* has been reported to have high activity towards aliphatic alcohols with short-chain (methanol, ethanol), but the activity decreases with increase of the chain length, although the oxidation of longer chains is also possible 64-66. This explains why methanol exhibits higher background than other alcohols. AOX usually exhibits higher activity for methanol than ethanol or other long-chain alcohols 67. But the introduction of the aerogel circumvents such a shortcoming by importing extra weak interactions between the enzyme and aerogel substrate, possibly including hydrophobic interactions and hydrogen bonding between -COOH and -OH groups on aerogel surface and methanol 68. Therefore, besides the role in biological sample collector, the presence of the aerogel also improves the specificity in alcohol tests. This also proves the rationality of the aerogel being as the sample collector.

Based on the above, the alcohol detection in saliva samples was realized. In general, the measurement of alcohol by commercial breathanalyzer is affected by many factors, such as local weather, alcoholic food and individual traits. Sometimes the breathanalyzer with fluctuating results requires repeated measurements. In our experiment, statistical analysis indicated positive results for six volunteers by breathanalyzer (Table 1). The saliva samples from six volunteers were collected and analyzed by our method. The results were all higher than the national standard for DUI (1.74 mM in saliva), indicating all the volunteers suffered from different levels of alcoholism, and two of them reached DWI level. The advantage of our method over the breathanalyzer is not only limited to more reliable detection results, but also includes the realization of quantitative determination of alcohol instead of qualitative detection.

### Detection glucose using 6-PAAC-30

**Figure 6:**
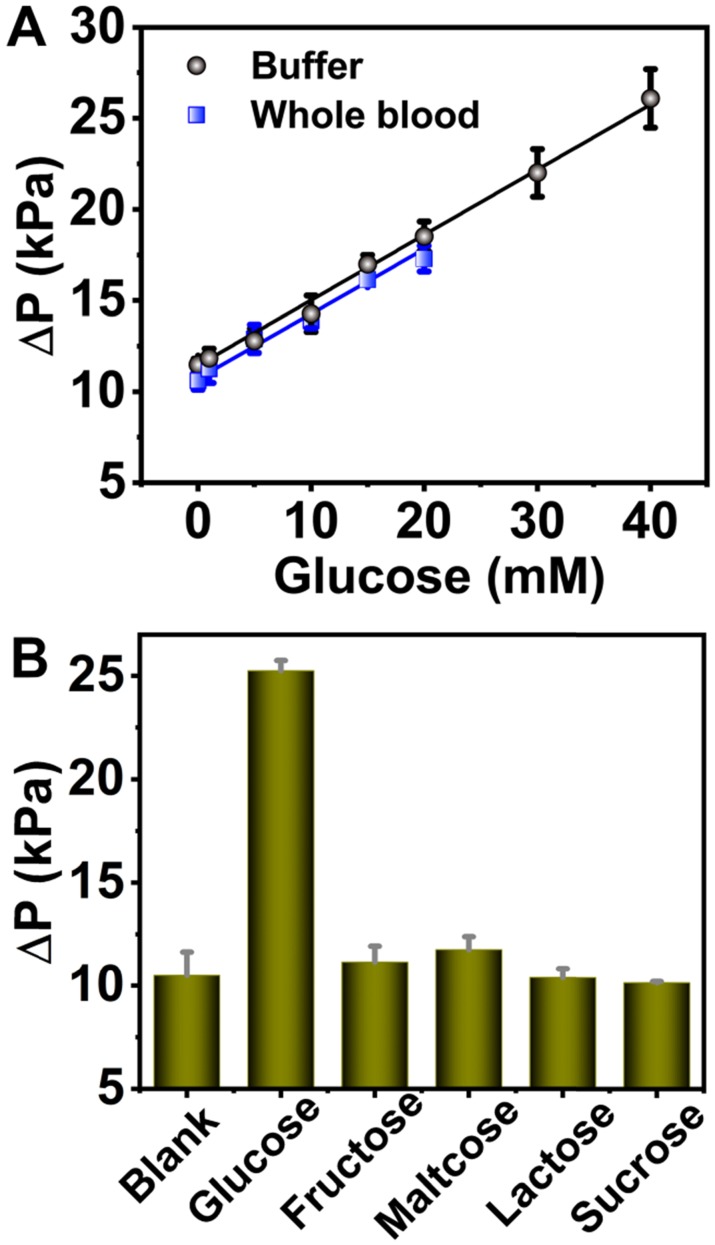
** (A)** The linear correlation between ΔP and glucose concentration in the 0-40 mM range in buffer (black dots) and 0-20 mM range in 0.5% whole blood (blue dots). **(B)** Selectivity analysis for glucose detection. The analyte concentrations were all 20 mM.

In order to demonstrate the universality of 6-PAAC-30, it was also employed to detect glucose in buffer solution as well as in blood sample, which can be transformed into O_2_ under the aid of GOX. ΔP values in buffer were linearly correlated with glucose concentrations of 0-40 mM, the regression equation was ΔP = 0.373 C_Glucose_ + 11.0 with R^2^ of 0.998. The LOD was calculated to be 2.50 mM. In 0.5% whole blood samples, an average recovery rate up to 99.2% was obtained (Figure 6A). Similar as the anti-interference tests above, fructose, maltose, lactose and sucrose were selected to evaluate the selectivity. Very weak signals were produced by the analogues thanks to the specificity of GOX to glucose (Figure 6B). The detection results in 0.5% whole blood were compared with the ones from commercial personal glucose meter (Table 2). It can be seen that the glucose recoveries fall in the range of 97.1%-106% by glucose meter, and 93.8%-102% by pressure meter. The results indicate that this method is comparable with personal glucose meter in accuracy, and is promising to be applied in glucose detection in real samples.

## Conclusions

In summary, we designed an amphiphilic aerogel with its surface modified with Pd@Pt core-shell nanoparticles. The aerogel functioned as a highly biocompatible sponge-like absorbent that can be used as biological sample collector and the anchored Pd@Pt core-shell nanoparticles contributed its excellent catalytic activities to the conversion of the analytes into gas pressure signals. The assay based on pressure readout induced by gas generation exhibited reliable diagnosis of alcohol with a detection limit of 0.50 mM, which is lower than the legal limit for driving. The measurements of saliva alcohol concentration by our method showed more reliable results and the ability in distinguishing DUI and DWI. In addition, the higher affinity between amphiphilic aerogel and methanol reduced the interference of alcohol sensing. The proposed sensor is considered as a promising alternative to blood alcohol detection in light of its noninvasive merit. This method is also compatible in glucose detection no matter buffer or whole blood samples with comparable accuracy with commercial glucose meter. The Pd@Pt-loaded aerogel exhibits good biocompatibility and the whole detection process produces no harmful emission. With the advantages of excellent generality, anti-interference ability, low cytotoxicity and environmental friendliness of the detection process, the design philosophy in our system may pave a new way in development of point-of-care assay for other analytes in biological system.

## Supplementary Material

Supplementary figures.

## Figures and Tables

**Table 1:** Comparison of the results of alcoholism testing in saliva by breathanalyzer and pressure meter.

Method	Volunteer 1	Volunteer 2	Volunteer 3	Volunteer 4	Volunteer 5	Volunteer 6
Breathanalyzer	+	+	+	+	+	+
Pressure meter	DUI/5.88*	DUI/2.94	DUI/4.41	DWI/7.98	DWI/13.0	DUI/1.89

+ means alcoholism testing is positive. * means saliva alcohol concentration was 5.88 mM.

**Table 2:** Comparison of the results of glucose detection in 0.5% whole blood by personal glucose meter and pressure meter.

Method	Spiked Glucose /mM	Concentration (mean±σ, n=3) / mM	Recovery /%
Glucose meter	5	5.31 ± 0.0907	106
	10	10.2 ± 0.349	102
	15	14.6 ± 0.192	97.1
Pressure meter	5	4.85 ± 0.212	96.3
	10	9.41 ± 0.141	93.8
	15	15.3 ± 0.208	102

The final recovery data were adjusted with 0.0322 mM glucose in 0.5% whole blood (the whole blood glucose was 6.44 mM detected by personal glucose meter).

## Abbreviations

POC: point-of-care
LOD: limit of detection
PVA: ploy(vinyl alcohol)
MA: maleic acid
PAA: PVA-based amphiphilic aerogel
CAT: catalase
POD: peroxidase
AOX: alcohol oxidase enzyme
GOX: glucose oxidase
PAAC: PAA/Pd@Pt composite
Pd@Pt: free-standing core-shell nanoparticles
ΔP: pressure change
LA: loading amount
DUI: driving under influence
DWI: driving while impaired
