# Supplementary material for: Point-of-care assay for drunken driving with Pd@Pt core-shell nanoparticles-decorated ploy(vinyl alcohol) aerogel assisted by portable pressure meter
Source: Theranostics. 2020 Apr 6;10(11):5064–73. doi: 10.7150/thno.42601 (PMC7163434; doi:10.7150/thno.42601)
Supplement: Supplementary file 1 — Supplementary figures. [file thnov10p5064s1.pdf]

Supporting Information

Point-of-care assay for drunken driving with Pd@Pt core-shell nanoparticles-ploy(vinyl alcohol) aerogel assisted by portable pressure meter

Yu Zhang,[1] Quanyi Liu, [1,2] Chong-Bo Ma,[1,3*] Qingqing Wang, [1] Meiting Yang,[1] Yan Du.[1,2*]

1. State Key Laboratory of Electroanalytical Chemistry, Changchun Institute of Applied Chemistry, Chinese Academy of Sciences, Changchun, Jilin 130022, China.
E-mail: duyan@ciac.ac.cn; duyan.bessie@gmail.com
2. Department of Chemistry, University of Science & Technology of China, Hefei, Anhui 230026, China
3. Key Laboratory of Nanobiosensing and Nanobioanalysis at Universities of Jilin Province, Key Laboratory of Polyoxometalate Science of Ministry of Education, National & Local United Engineering Laboratory for Power Batteries, Department of Chemistry, Northeast Normal University, Changchun, Jilin Province 130024, P.R. China
Email: macb806@nenu.edu.cn; chongbo.ma@gmail

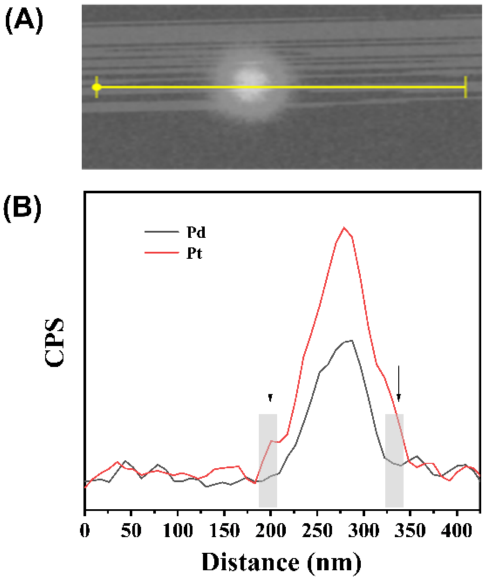

Figure S1. (A) The image of target nanoparticle and scan path. (B) EDS line scans of Pd and Pt recorded from a Pd@Pt core-shell nanoparticle. The two regions in gray indicate the ~20 nm thickness of the Pt shell.

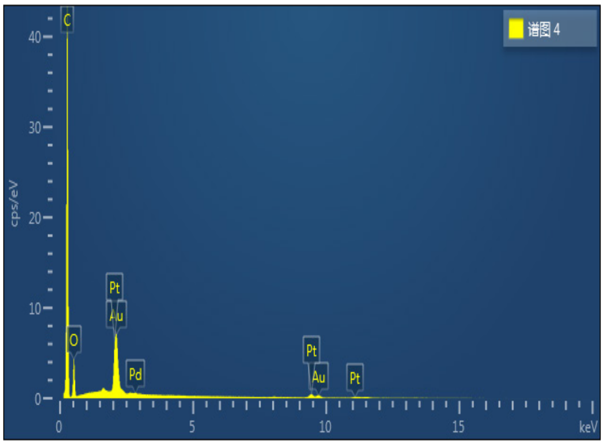

Figure S2. EDX spectroscopy analysis of 6-PAAC-30.

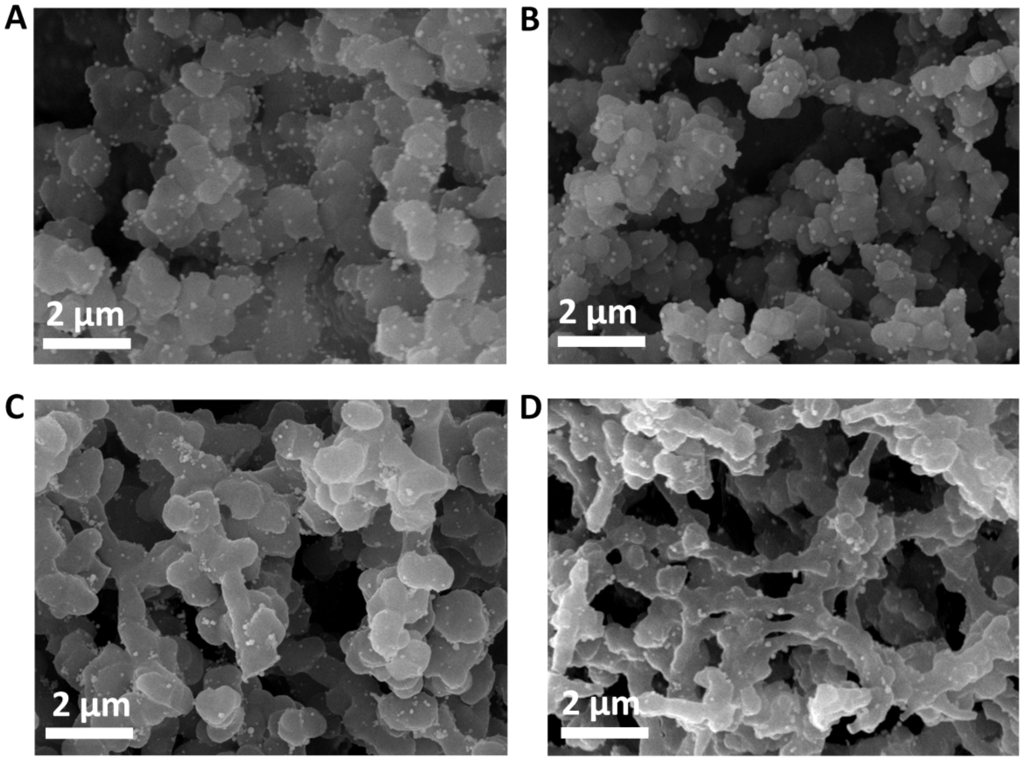

Figure S3. SEM images of (A) 5-PAAC-40, (B) 6-PAAC-40, (C) 7-PAAC-40 and (D) 8-PAAC-40.

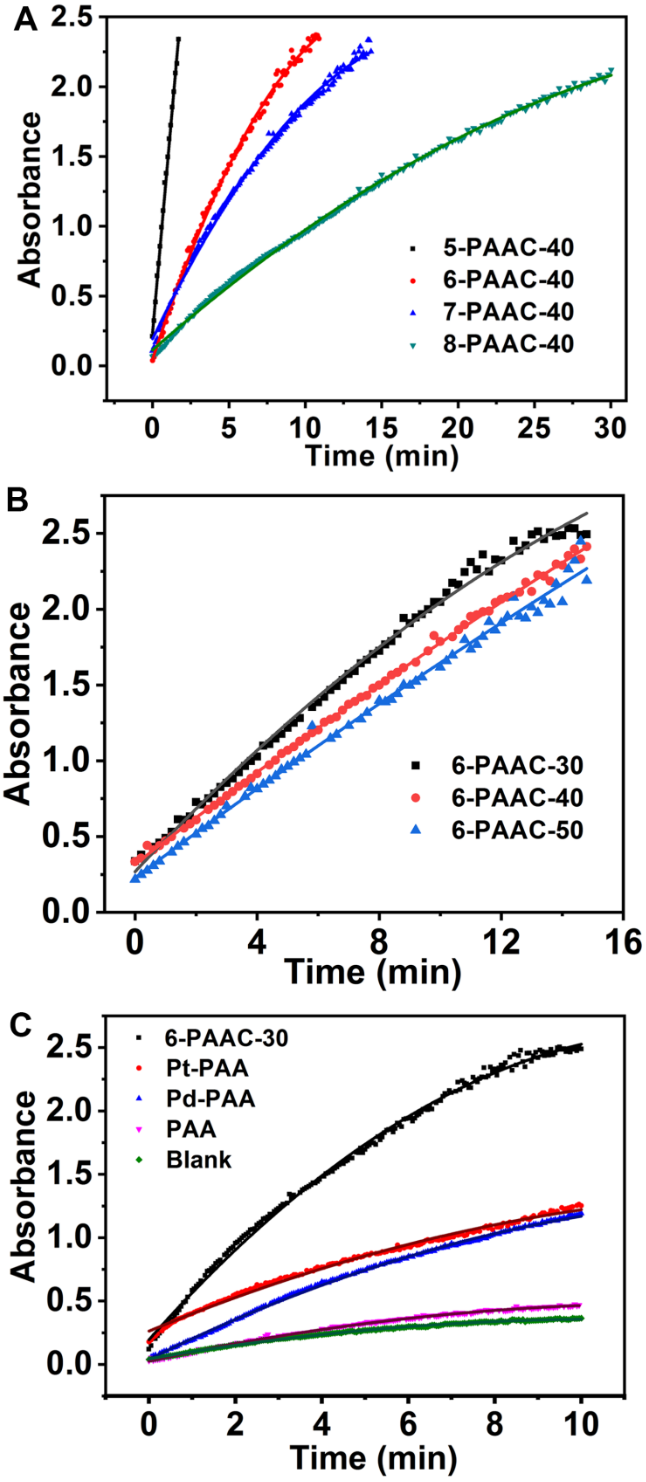

Figure S4. Time-dependent absorbance changes of TMB at 652 nm varied with different catalysts used for testifying the POD activity. Experimental conditions: MES-AC buffer solution, pH 4.5; TMB, 0.8 mM; H_2O_2, 1.0 mM; catalysts, 1.0 mg; measured at 37 ^{o}C.

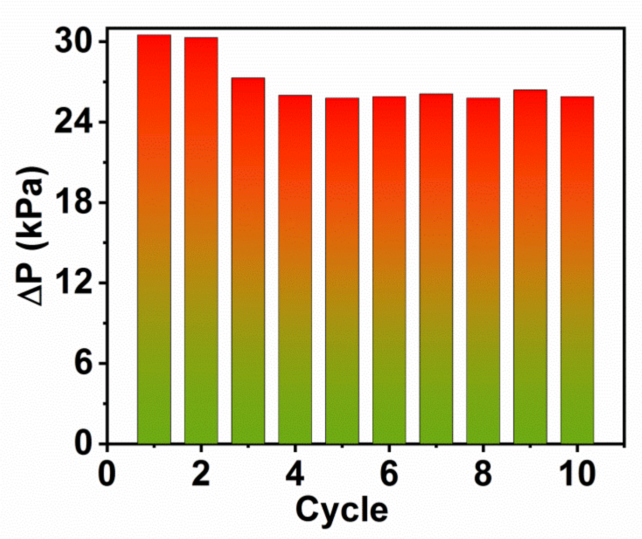

Figure S5. Reuse of 6-PAAC-30 (1.0 mg) in 20 mM H_2O_2 decomposition.

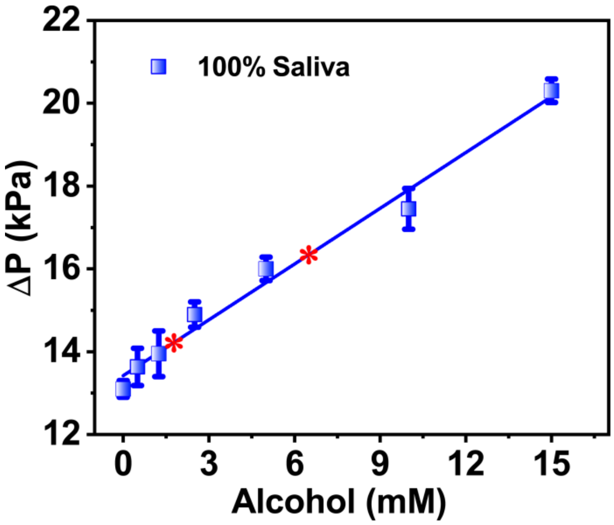

Figure S6. The linear correlation between ΔP and alcohol concentration in 100% saliva calculated from the curve in 40% saliva sample (blue line of Figure 4A, in manuscript). Red snowflakes represent DUI and DWI, respectively.
